# Digital Health Interventions for Type 2 Diabetes: A Narrative Review of Mobile Technologies and Their Impact on Patients' Outcomes

**DOI:** 10.1002/hsr2.72116

**Published:** 2026-05-25

**Authors:** Farnam Gohardehi, Behdad Ghanami, Melika Silakhor, Pooria Sobhanian

**Affiliations:** ^1^ Student Research Committee, Faculty of Medicine Mazandaran University of Medical Sciences Sari Iran

**Keywords:** mhealth, mobile applications, telemedicine, type 2 diabetes mellitus

## Abstract

**Background and Aims:**

Type 2 diabetes mellitus (T2DM) is an increasing worldwide health issue that carries considerable economic implications. Traditional management approaches often fail to provide continuous support between clinical visits, creating gaps in care that lead to suboptimal outcomes. Mobile health (mHealth) innovations have become effective solutions to address these challenges through real‐time monitoring and personalized guidance. Therefore, we reviewed the efficacy of mobile health technologies in improving the outcomes of patients with T2DM.

**Methods:**

This narrative review was conducted by searching PubMed from January 2015 to January 2025, using Keywords such as Type 2 Diabetes Mellitus, Telemedicine, mHealth, and mobile applications.

**Results:**

Out of 1356 studies, 36 studies met the criteria for final evaluation. Mobile health innovations hold significant promise for enhancing both clinical and patient‐reported outcomes. These technologies have been shown to positively impact clinical parameters such as glycemic control and cardiovascular risk factors, as well as patient‐reported outcomes such as self‐efficacy and quality of life.

**Conclusion:**

Despite growing evidence supporting mHealth clinical utility among patients with T2DM, mobile health solutions still face implementation barriers, including data processing challenges and limited clinical integration.

## 1 Introduction

Type 2 diabetes mellitus (T2DM) is a chronic condition with growing incidence globally, driven by lifestyle changes, aging populations, and urbanization [1, 2]. It is linked to severe complications, such as cardiovascular disease, kidney failure, and neuropathy, posing significant public health and economic challenges [1, 3]. Managing T2DM requires consistent monitoring of glycemic control, medication adherence, and lifestyle modifications [1, 4]. However, traditional care models often fail to provide continuous support between clinical visits, leaving patients vulnerable to poor disease management [4]. This lack of continuous support is especially critical in low‐ and middle‐income countries (LMICs), where inadequate resources, low digital and health literacy, and socioeconomic barriers restrict effective diabetes management [5]. Consequently, there is a demand for novel strategies to empower patients in self‐management. Mobile health (mHealth) technologies serve as valuable tools to address these challenges by delivering real‐time monitoring and personalized guidance that complement conventional care [1].

Over the past decade, mHealth interventions have advanced significantly, encompassing smartphone applications, wearable devices, and continuous glucose monitoring systems [6, 7]. These technologies provide functionalities such as glucose tracking, dietary advice, physical activity monitoring, and medication reminders [7]. Preliminary evidence suggests that mHealth interventions improve clinical outcomes like HbA1c levels and cardiovascular risk factors while enhancing patient‐reported outcomes such as self‐efficacy and quality of life [7, 8, 9]. Despite their potential, integrating these tools into standard care remains limited due to data processing and adoption challenges. To the best of our knowledge, this is the first narrative review to comprehensively synthesize the impact of diverse mobile health technologies on clinical and patient‐reported outcomes in T2DM, while addressing intervention types and implementation challenges. Therefore, we assessed how mobile health technologies improve the results for individuals with T2DM. By synthesizing current evidence, this review contributes valuable insights to existing knowledge and highlights the capability of mHealth interventions in improving patient outcomes and optimizing healthcare efficiency. Additionally, it helps identify the strengths and limitations of these technologies in real‐world settings while offering practical guidance for clinicians seeking to integrate digital health solutions into diabetes care.

## 2 Methods

**FIGURE 1:**
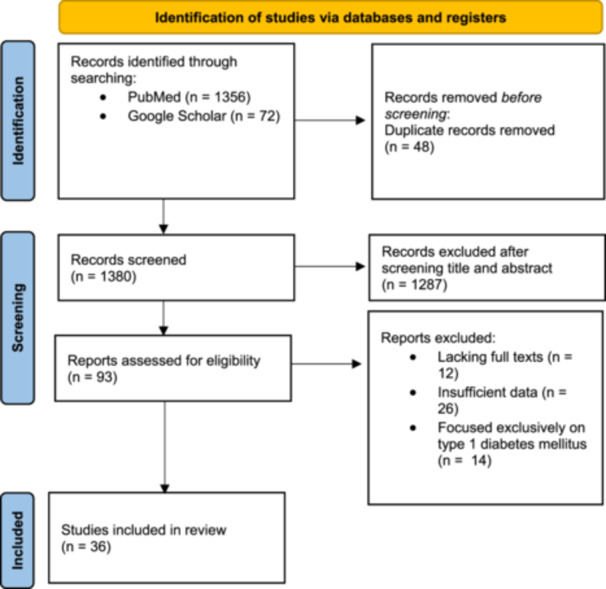
PRISMA flow diagram of the review process.

This study was a narrative review. The PubMed database and Google Scholar search engine were searched between January 2015 and January 2025 using Keywords such as “Type 2 Diabetes Mellitus,” “NIDDM,” “Telemedicine,” “Digital Health,” “Mobile Application,” “Mobile Phone,” “Smartphone,” and “mHealth.” Key journals were manually searched, and reference lists from selected studies were reviewed for further relevant research. The search strategy for the PubMed database is provided in Supporting Information Table S1. Studies focusing on the management of T2DM through digital health interventions, specifically mHealth, telemedicine, and mobile applications, were included in the review. Eligible studies needed to report clinical outcomes (such as HbA1c levels and blood pressure) and patient‐reported outcomes (such as quality of life or self‐efficacy). Included study types comprised randomized controlled trials, cohort studies, feasibility studies, and observational studies reporting these outcomes. Studies lacking abstracts, full texts, or sufficient relevant data were excluded from the review process. Studies focusing exclusively on type 1 diabetes mellitus were also excluded. Figure 1 demonstrates the process of searching, screening, and selecting studies for this review. Three authors (F.G., B.Gh., and M.S.) performed the screening process and checked the eligibility criteria based on the title and abstract. Full texts of selected studies were then evaluated, and any disagreements were resolved by the third author (F.G.). Data extraction included the author's name, publication year, study location, study design, study population, study tool, modality/intervention type, study outcomes, key findings, and study limitations. Because of the substantial heterogeneity among the included studies, the results were synthesized qualitatively.

## 3 Results

Out of 1428 retrived records from literature searching, 36 studies were included in the review for qualitative synthesis. The features of the included studies are presented in Table 1. These studies assessed the efficacy of mobile health technologies on clinical outcomes, including glycemic control, cardiovascular risk factors, insulin dosage, and diabetes complications, as well as patient‐reported outcomes, such as self‐efficacy and quality of life.

**TABLE 1:** Characteristics of included studies.

Authors	Year	Location	Study design	Population	Tool/Intervention	Modality/intervention type	Study outcome (s)	Key findings	Study limitations
Waki et al. [25]	2024	Japan	RCT	T2DM patients with UACR = 30–299 mg/g creatinine *n* = 66 Intervention *n* = 60 Control	DialBetesPlus app	Smartphone application measuring daily step counts, blood glucose, blood pressure, body weight and providing feedback on exercise and diet, based on TPB	UACR, HDL cholesterol, HbA1c	A 12‐month mHealth intervention significantly improved UACR and HbA1c in diabetic kidney disease patients, with exercise being the key factor.	Lack of blinding
Guo et al. [14]	2023	China	RCT	T2DM patients aged 18–75 years with no cognitive impairments and the ability to understand mHealth management procedures *n* = 32 Intervention *n* = 32 Control	Implantable glucose sensor	Mobile app (providing dietary, physical and health education program), network platform, implantable glucose sensor, GP support	BMI, FBG, 2hPP, HbA1c, PRO	A 4‐week mHealth intervention using an implantable glucose sensor and general practitioner‐guided mobile app significantly improved glycemic control (FBG, 2hPG, HbA1c), reduced BMI, and enhanced quality of life and self‐management in Chinese T2DM patients.	Small sample size, Single‐site recruitment
Hermanns et al. [23]	2023	Germany	RCT	T2DM patients, BMI ≥ 25.0 kg/m², HbA1c > 7.5% and < 10.0%, mean age = approximately 60 years, and qualification for basal insulin treatment (with oral/non‐insulin injectables) *n* = 119 Intervention *n* = 107 Control (written titration schedule for basal insulin)	MDC	MDC app (calculating basal insulin dose using 3 consecutive fasting blood glucose levels, text messaging)	HbA1c, FBG, Insulin Dosage	MDC app achieved significantly greater HbA1c reduction compared to written titration charts in T2DM patients.	Short trial duration
Lee et al. [10]	2023	South Korea	RCT	T2DM patients aged 19–69 years, HbA1c 7.0%–8.5%, BMI ≥ 23.0 kg/m^2^, Stable dose of oral antidiabetic agents for > 12 weeks or no antidiabetic agents for 4 weeks. *n* = 99 Group A (Usual care) *n* = 97 Group B (Digital health) *n* = 98 Group C (Digital health + feedback from medical professionals + intermittent personal CGM)	FoodLens Program	Foodlens program (AI food recognition for automated dietary logging), CGM (Dexcom G5), personalized feedback through text messages, Bluetooth‐connected devices = glucometer, sphygmomanometer, scale with bioelectrical impedance analysis, pedometer, dietary interventions	HbA1c, FBG, Body Weight	An AI‐powered digital health platform significantly improved long‐term blood glucose control (0.44% HbA1c reduction) and weight management in T2DM patients.	Different hypoglycemic events detection
Unnikrishnan et al. [24]	2022	India, Mexico, Colombia	Cohort	T2DM patients, mean age = 52.8 years, registered for MDC between August 1, 2018, and April 30, 2020 *n* = 2517 Intervention	MDC	MDC app (designed for basal insulin titration based on user‐input FBG and hypoglycemia data)	FBG, Insulin Dosage	Frequent MDC use ( ≥ 3x/week) boosted FBG target achievement by 44% and reduced time‐to‐target without increasing the risk of hypoglycemic events.	Retrospective design limits, High attrition
Lee et al. [15]	2022	South Korea	RCT	T2DM patients aged 19–74 years with a baseline HbA1c level of ≥ 7.5% and BMI of ≥ 18.5 kg/m², not being on insulin pump or pregnant. *n* = 71 UC *n* = 79 MC *n* = 84 MPC	iCareD system app	iCareD mobile app (presenting SMBG, dietary habits and step count), text messaging, personalized feedback every 2 weeks (MPC group), glucometer (all groups)	HbA1c, LDL cholesterol, PRO	A diabetes app with doctor feedback reduced HbA1c levels better than usual care at 3 months, especially in younger, overweight patients, though effects faded by 6 months. A total of 87.2% of users reported satisfaction with the app.	Lack of blinding, No medication adjustment
Tamez‐Pérez et al. [37]	2022	Mexico	Cohort	T2DM patients aged 18–75 years, HbA1c > 7%, owning a smartphone and being treated with any basal insulin *n* = 158 Intervention	MDC	MDC mobile app (basal insulin titration with personalized dose adjustment based on SMBG and hypoglycemia data), glucometer (FreeStyle Neo by Abbott USA)	HbA1c, FBG, Insulin Dosage, PRO	The MDC mobile app significantly improved glycemic control in T2DM patients, achieving target glucose ranges, reducing HbA1c by 1.78%, and enhancing well‐being scores, without severe hypoglycemia events during the 16‐week intervention.	No control group, Short trial duration
Hu et al. [38]	2022	China	Feasibility study (pre‐experimental)	Chinese immigrants with T2DM aged 18–70 years, understanding Mandarin Chinese, owning a smartphone/tablet, with current WeChat use *n* = 30 Intervention	DSME	Culturally tailored DSME videos (24 brief videos, ~5 min each) via WeChat, 2 video links weekly = 1 diabetes education video + 1 behavioral skills video, bimonthly support calls (15 min)	HbA1c, PRO	WeChat‐delivered diabetes education program demonstrated high feasibility (92% video engagement) and acceptability (9.9/10 satisfaction) while significantly improving HbA1c (−0.5%), self‐efficacy, and lifestyle behaviors.	Small sample size, No control group
Jiwani et al. [33]	2022	USA	Feasibility study (pre‐experimental)	Older overweight/obese adults (age ≥ 65 years, BMI ≥ 25 kg/m²) with T2DM *n* = 18 Intervention	Fitbit Wristband Activity Tracker	Fitbit wristband activity tracker application (designed for self‐monitoring, 10 group sessions for the behavioral lifestyle intervention, tailored weight loss (5%–7%) and physical activity goals (175 min/week)), fitbit wristband activity tracker	Body Weight, BMI, PRO	Fitbit app produced significant reduction in frailty scores and body weight in predominantly pre‐frail/frail older adults with T2DM.	Small sample size, No control group
Vaughan et al. [21]	2022	USA	RCT	Spanish‐speaking low‐income latinos with T2DM aged ≥ 18 years with a baseline HbA1c of ≥ 6.5% *n* = 101 Intervention	TIME Program	Monthly 3‐h community health worker‐led group visits with weekly telehealth/mHealth support for glucose monitoring and medication adherence (Intervention months 1–6), usual clinic care, with mHealth support during months 7–12. No mHealth support in months 13–24 (Post‐Intervention months 7–24)	HbA1c	Low‐income Latinos with T2DM maintained significant HbA1c reductions through 24 months of the TIME intervention.	High attrition, Missing HbA1c data
Christensen et al. [19]	2022	Denmark	RCT	T2DM patients aged 18–70 years, BMI 30–45 kg/m^2^ *n* = 100 Intervention *n* = 70 Control	LIVA eHealth App	Liva 2.0 mobile app, digital health coaching, goal setting for nutrition, physical activity, and sleep	Body Weight, BMI, HbA1c, Lipid Profile (total cholesterol, LDL, HDL, triglycerides)	The eHealth lifestyle coaching program achieved significantly greater weight loss and HbA1c normalization compared to standard care in T2DM patients at 6 months.	High attrition, Short trial duration
Bults et al. [35]	2022	Netherlands	Explanatory sequential	T2DM patients aged ≥ 16 years *n* = 103 (completed the questionnaire)	Web‐based questionnaire	Filling out a questionnaire to evaluate barriers and drivers among users and nonusers of T2DM apps	Barriers and Limitations	Involvement of healthcare providers is essential to help T2DM patients effectively use apps.	Selection Bias, Moderate sample size
Esferjani et al. [30]	2022	Iran	RCT	Elderly (aged ≥ 60 years) with T2DM communicating in Persian, without cognitive impairments, owning a cellphone *n* = 59 Intervention *n* = 59 Control	Educational content via WhatsApp and booklet	Three online training sessions via WhatsApp designed for groups (each containing 10–11 participants) lasting 60 min.	PRO, HbA1c	A mobile‐based educational intervention significantly improved self‐care behaviors, psychosocial factors, and HbA1c in elderly patients with T2DM.	Single‐site recruitment, Not studied in younger adults
Krishnakumar et al. [39]	2021	India	RCT	T2DM patients aged ≥ 18 with an initial HbA1c of > 6.5%, owning an Android smartphone and active internet connection, without a history of a major surgical procedure during the past 6 months and absence of any medical condition impairing the ability to walk for 15–30 min daily *n* = 102 Intervention	Wealthy CARE app	Wealthy CARE mobile app (integrating data logging for blood glucose, meals, physical activity, and weight), personalized feedback with the help of an AI‐powered chatbot,	HbA1c, FBG, 2hPP, Body Weight, BMI	The Wellthy CARE digital therapeutic significantly improved glycemic control (HbA1c, FBG, PPBG), weight, and BMI in South Asian patients with T2DM.	No control group, Short trial duration, Gender Imbalance
Batch et al. [40]	2021	USA	Cohort	T2DM patients aged 18–89 years with an HbA1c level of ≥ 8% and < 12% in the previous 3 months, and access to an iOS or Android smartphone *n* = 100 Intervention *n* = 101 Control	Time2Focus	Time2Focus mobile app (12‐week, level‐based skill‐building program focusing on diet, exercise, and glucose monitoring, designed to enhance patient confidence and skill in T2DM management)	PRO	The Time2Focus app enhanced self‐efficacy in engaged T2DM patients, though HbA1c changes weren't significant.	Small cohort, Short duration of the study, High Attrition
Martin et al.[31]	2021	USA	Cohort	T2DM patients aged ≥ 18 years, English fluency, and consent capability *n* = 226 Intervention	Pack Health program	Digital health coaching, 3–5 weekly SMS text messages/emails, exercise band, goal magnet, educational video lessons	HbA1c, BMI, Body Weight, PRO	Pack Health program proved most effective for high‐risk T2DM patients, delivering the greatest improvements in blood glucose control and overall well‐being, while also benefiting participants across all risk levels through personalized support.	No control group, Small cohort
Zimmermann et al. [41]	2021	USA	Retrospective study (case series)	T2DM patients aged ≥ 18 years, with baseline HbA1c ≥ 7.0%, owning a smartphone or web access, fluent in English or Spanish *n* = 258 Intervention	Vida Health app	Vida Health mobile app (providing live audio‐video sessions, text messaging, SMBG, food intake and activity tracking)	HbA1c	Digital diabetes management delivered clinically significant HbA1c reductions (mean −0.81%, *p* < 0.001), with high‐risk patients (baseline HbA1c ≥ 8%) showing greater improvements ( − 1.44%) and higher program engagement.	Lack of randomization, High Attrition
Li et al. [42]	2021	China	RCT	Patients aged 18–64 years, with access to a smartphone and being diagnosed with T2DM (oral glucose tolerance test) within the last 10 years *n* = 44 Intervention *n* = 41 Control	R plus Health	R plus health app, wireless chest‐worn heart rate band, exercise programs, exercise testing (resting heart rate, step test and muscular endurance)	Body Fat Percentage	Remote monitoring with a fitness app and heart rate band improved cardiopulmonary endurance and reduced body fat more effectively than self‐reported exercise in T2DM patients.	Baseline age and hypertension differences between groups
Yasmin et al. [43]	2020	Bangladesh	RCT	T2DM patients receiving treatment and owning a smartphone *n* = 142 Intervention (mean age = 53 years) *n* = 131 Control (mean age = 51 years)	Mobile health intervention	Personalized voice calls (supporting diet, medication, physical activity) every 10 days, 24/7 physician‐staffed call center.	PRO	A mobile health intervention significantly improved adherence to diabetes management, including diet, exercise, and tobacco cessation.	Single‐site recruitment, Gender imbalance, Attrition
Kumar et al. [44]	2020	India	RCT	Adults diagnosed with T2DM for at least 1 year, aged 18–65 years, being treated with insulin and/or oral drugs, owning an Android smartphone *n* = 150 Intervention *n* = 150 Control	DIAGURU	Android smartphone, “DIAGURU” mobile application (providing blood sugar and insulin level chart, computing food caloric value)	PRO	After 6 months, the intervention group showed a significantly better improvement in quality of life compared to the control group.	Not calculating the sample size scientifically
Gong et al. [12]	2020	Australia	RCT	T2DM patients aged ≥ 18 years with basic English skills and access to an internet‐enabled smart device *n* = 93 Intervention *n* = 94 Control	MDC	MDC mobile app (featuring an embodied conversational agent (“Laura”) for personalized coaching), a printed user guide, a supplemental website with forums, an optional Bluetooth glucose meter, and program coordinator support	PRO	The MDC program enhanced health‐related quality of life in T2DM patients, though did not significantly lower HbA1c.	Lack of blinding, small sample size
Young et al. [32]	2020	USA	RCT	T2DM patients aged ≥ 18 years, receiving care at one of three Northern California primary care clinics (two suburban, one urban), HbA1c ≥ 6.5% *n* = 132 Intervention *n* = 155 Control	P^2^E^2^T^2^ program	Nurse health coaching (6 sessions) by motivational interviewing‐based registered nurses, mHealth technology, including wearable device (Basis Peak and Garmin VivoSmart Heart Rate) to track steps, heart rate, sleep, MyFitnessPal app (optional nutrition logging)	PRO	A nurse‐led mHealth coaching program significantly improved short‐term diabetes self‐efficacy and reduced depressive symptoms in T2DM patients, though effects diminished by 9 months despite sustained increases in physical activity.	Setting dependent, High Resource Requirements
Li et al. [45]	2020	China	Cohort	T2DM, mean age = 52.24 years, mean HbA1c level = 7.76%, 60% male and 40% female *n* = 1200 mHealth group *n* = 1200 Control (Usual care)	Mobile‐based intervention	Mobile app, smart wearable devices (e.g., wireless glucose monitor and blood pressure monitor), web platform. Data‐sharing cloud platform	HbA1c, FBG, 2hPP	Mobile‐based intervention enhances glycemic control rates.	Retrospective Design Limit, Incomplete Clinical Records, Missing Values
Lazo‐Porras et al. [27]	2020	Peru	RCT	T2DM patients aged between 18 and 80 years, being in risk group 2 or 3 based on the diabetic foot risk classification system, palpable dorsalis pedis pulse in both feet, owning a mobile phone *n* = 86 Intervention *n* = 86 Control	TempStat thermometry device and mHealth reminders	TempStat device (generating a visual representation of the temperature data, for both intervention and control arm), mHealth component (featuring 2 weekly reminder messages and 6 weekly foot‐care promotion messages through SMS and voice messaging)	DFN	The addition of mHealth reminders to foot thermometry did not reduce DFU incidence in high‐risk patients, with a 24% cumulative ulcer rate in the intervention group vs. 11% in the thermometry‐only control group.	Higher DFU risk in the intervention group, Lower adherence
Sokolovska et al. [26]	2020	Latvia	RCT	T2DM patients aged 35–75 years, without previous cardiovascular disease or high cardiac risk/chronic kidney disease stage IV–V/severe diabetic eye complications/severe peripheral neuropathy or musculoskeletal impairment *n* = 14 Intervention *n* = 26 Control	Instawalk app	Instawalk mobile app delivering 60‐min training sessions (including 3‐min intervals at 40% and 70% peak intensity), 3 sessions per week training for 4 months, Polar H10 heart rate monitors for intensity control.	Albuminuria, Leptin/Adiponectin Ratio	Interval walking via smartphone app improved vascular health in T2DM by reducing albuminuria and leptin/adiponectin ratio.	No dietary control, No interim VO₂ testing, No body composition measurements
Majithia et al. [17]	2020	USA	RCT	T2DM patients aged ≥ 18 years with a baseline HbA1c of ≥ 8%–≤ 12%, consented to use a blood glucose meter and CGM, and smartphone ownership *n* = 55 Intervention	Onduo VDC	Mobile app, personalized coaching, connected devices, including blood glucose meters and Dexcom G6 RT‐CGM, video consultations	HbA1c, SBP, Lipid Profile (total cholesterol, LDL, triglycerides)	The Onduo VDC demonstrated significant improvements in HbA1c, weight loss and metabolic markers over 4 months.	Cross‐sectional design limits
Sean Duffy et al. [46]	2020	Guatemala	Cohort	T2DM patients aged ≥ 18 years *n* = 67 Intervention	CommCare Platform	Smartphone application with algorithmic clinical decision support, community health care workers (trained for 15 h in multiple sessions, educating diabetes care	HbA1c, Renal Function	A community health worker‐delivered diabetes program supported by a smartphone app significantly reduced HbA1c levels and doubled the proportion of patients achieving glycemic control in rural Guatemala.	No control group, Small sample size, Gender imbalance
Koot et al. [20]	2019	Singapore	Feasibility study	T2DM patients aged 21–70 years, HbA1c ≥ 7.5% for the past 2 months, BMI > 23 kg/m^2^, not being on insulin, owning an iPhone or Android mobile phone *n* = 100 Intervention	Glycoleap app	Glyco app, glucometer kit, BodyTrace wireless weightning scale, resistance band, online health sessions, physical activity tracking, meal logging	HbA1c, Body Weight	Glycoleap program improved HbA1c levels with high user satisfaction.	Short trial duration, No control group, Low participation rate
Jeffrey et al. [36]	2019	Australia	Qualitative study	T2DM patients aged ≥ 18 years from rural Australian communities, owning a smartphone *n* = 16 app users (Intervention) *n* = 14 non‐app users (Control)	Diabetes Journal Apps	Semi‐structured interviews for mobile app users and non‐users	PRO	Although intuitive tracking features in diabetes apps improved self‐management outcomes, broader adoption requires addressing key barriers, including clinician participation gaps and patient education.	Small sample size, No app usage data
Nepper et al. [47]	2019	USA	Quasi‐experimental study	T2DM patients aged ≥ 30 years, HbA1c > 6.5%, owning a cellular phone with text messaging capability *n* = 40 Intervention *n* = 39 Control	Text Messaging Intervention	Text messaging (3 educational text messages per week for 12 weeks, sent between 11 a.m. and 2 p.m., covering topics, including nutrition, physical activity, diabetes self‐management, medication adherence, and risk of diabetes complications)	PRO, CVD Awareness	A 12‐week text message program significantly improved CVD risk awareness, availability of healthy food, and physical activity in T2DM patients.	Short trial duration, Non‐randomized design
Yu et al. [48]	2019	China	RCT	T2DM patients aged 35–65 years, owning a mobile phone *n* = 47 Group A (no MPA, no SMBG) *n* = 45 Group B (SMBG only) *n* = 48 Group C (MPA only) *n* = 45 MPA and SMBG (group D)	Diabetes‐Carer Mobile Application	Diabetes‐Carer mobile application, blood glucose meter, diabetes education (diet library with glycemic index, exercise videos/pictures, medical knowledge, diabetes self‐management, text messages, voice messaging	HbA1c	Diabetes‐Carer app significantly increased the proportion of patients achieving HbA1c < 7% after 24 weeks, indicating greater effectiveness than blood glucose self‐monitoring alone.	Unmeasured behavioral factors, Exclusion of elderly patients
Kim et al. [49]	2018	South Korea	RCT	T2DM patients aged 19–80 years, HbA1c 7.0%–10%, stable glycemic control for at least 3 months *n* = 90 Intervention (mDiabetes group) *n* = 82 Control (pLogbook group)	mDiabetes system	mDiabetes application (developed for insulin dosing), Bluetooth‐enabled glucometer (both groups), activity tracker (mDiabetes group only)	HbA1c, FBG, PRO Body Fat Percentage	The mDiabetes smartphone system outperformed traditional logbooks, helping more patients achieve better blood glucose control safely, with twice as many reaching ideal HbA1c levels under 7% compared to the paper logbook group.	Small subgroup sample size, Short study duration, Age difference among subgroups
Berman et al. [50]	2018	USA	Cohort	T2DM patients aged 18 years or older with an initial HbA1c > 6.5%, owning an Android or iPhone smartphone, 81.4% female, mean age = 50.7, mean BMI = 38.1, mean HbA1c = 8.1% *n* = 118 Intervention	Farewell	Mobile app with human support, recommending dietary patterns, meal planning and physical exercise, weight self‐monitoring via digitally connected device	HbA1c, PRO	Farewell digital therapeutic significantly lowered HbA1c levels, with greater glycemic control linked to higher app engagement.	No control group, Short trial duration
Kleinman et al. [11]	2017	India	RCT	Adults aged 18–65 years with T2DM ( > 6 months duration), HbA1c of 7.5%–12.5%, stable diabetes treatment, owning an Android smartphone, fluent in English/Hindi/Gujarati/Tamil *n* = 44 Intervention *n* = 47 Control	Gather Health	Gather app (based on theories of behavior change, e.g., health belief model and self‐efficacy, in‐app messaging, task reminders)	HbA1c, PRO	Gather health intervention significantly improved glycemic control, medication adherence, and blood glucose self‐testing frequency compared to usual care, with high engagement and satisfaction.	Lack of blinding, Short trial duration
Fortmann et al. [18]	2017	USA	RCT	Low‐income Hispanic patients with T2DM aged 18–75 years with a baseline HbA1c of ≥ 7.5%, *n* = 63 Intervention *n* = 63 Control	Dulce Digital Text Messaging Intervention	Text messaging, blood glucose meter (OneTouch Verio Meter; LifeScan, Inc., Milpitas, CA), questionnaire, motivational and medical support through messages	HbA1c, PRO	A texting program successfully managed T2DM in Hispanic patients, offering a low‐cost option for low‐income countries.	Lack of blinding, Short trial duration
Peng et al. [34]	2016	USA	RCT	Patients aged 18–70 years, owning a smartphone with an average diagnosis of T2DM for 8 years, 77.78% from rural areas, 44.44% with college or graduate degrees, and 66.67% having a health app on their mobile phones *n* = 18 Intervention	Workshop on health apps	Questions about users' app usage and knowledge, and reasons to like/dislike the app, 30‐min workshop demonstrating the self‐management features of four apps: GlucoseBuddy, mySugr, MyFitnessPal, and MapMyWalk	Barriers and Limitations	Rural T2DM patients found mobile apps potentially helpful for T2DM management but highlighted usability challenges and desired more engaging features to sustain consistent use.	Small sample size

Abbreviations: BMI, body mass index; CGM, continuous glucose monitoring; CVD, cardiovascular disease; DFN, diabetic foot neuropathy; DSME, diabetes self‐management education; FBG, fasting blood glucose; HDL, high‐density lipoprotein; LDL, low‐density lipoprotein; MC, mobile diabetes self‐care; MDC, my diabetes coach; MPC, mobile diabetes self‐care with personalized bidirectional feedback from physicians; PRO, patient‐reported outcome; RCT, randomized controlled trial; SBP, systolic blood pressure; SMBG, self‐monitoring of blood glucose; T2DM, type 2 diabetes mellitus; TPB, theory of planned behavior; UACR, urine albumin‐reatinine ratio; UC, usual care; 2hPP, two‐hour postprandial blood glucose.

### 3.1 Clinical Outcomes

#### 3.1.1 Glucose Control

- HbA1C
  The HbA1c test represents the average blood sugar level across about 3 months, corresponding to the lifespan of red blood cells. A randomized controlled trial (RCT) conducted in 2023 included 294 patients with T2DM, who were randomly assigned to three groups [10]. Group A received routine diabetes care every 3 months, serving as the control group, while Groups B and C utilized the “Auto‐Chek Care” digital healthcare platform. Participants in Group C received guidance from medical staff on how to use the platform, whereas those in Group B did not receive such support. Additionally, the FoodLens program enabled users to capture images of their food, automatically incorporating dietary data into the platform. Group C also received a continuous glucose monitoring (CGM) device and personalized feedback. The reductions in HbA1c levels from baseline to 24 and 48 weeks were significantly greater in Groups B (20.32% ± 0.58% to 24 weeks and 20.28% ± 0.56% to 48 weeks) and C (20.49% ± 0.57% to 24 weeks and 20.44% ± 0.62% to 48 weeks) compared to Group A, indicating the ability of the digital platform in advancing glycemic management. The Gather Health platform was evaluated in an RCT for its effectiveness as a mHealth intervention [11]. This platform featured a mobile app designed to offer guidance on behavior change, self‐care support, and alerts for urgent issues. Over 6 months, participants in the intervention group achieved an average HbA1c reduction of 1.5%, while the control group saw a decrease of only 0.8%, reflecting a statistically significant difference (*p* = 0.02).
  The “My Diabetes Coach” (MDC) program was evaluated in a 12‐month RCT involving 187 patients with T2DM, who were randomly divided into intervention (*n* = 93) and control (*n* = 94) groups [12]. The intervention group received coaching from a conversational agent named “Laura,” who monitored various aspects of diabetes management, including blood glucose levels, diet, physical activity, medication adherence, and foot care. In contrast, the control group received routine clinical treatment. Despite the supportive role of Laura and the program's algorithms designed to guide behavioral changes, HbA1c changes did not differ significantly between the intervention and control groups ( − 0.04; 95% CI − 0.45 to 0.36; *p* = 0.83).
  While HbA1c remains essential for glycemic evaluation, most mHealth studies have been limited to single‐center designs with small sample sizes. Among 23 studies demonstrating the positive impact of mHealth tools on HbA1c reduction, 16 included populations exceeding 100 participants, and 1 study involved a cohort of over 1000 individuals. To further establish the efficacy of mHealth interventions, there is a pressing need for comprehensive multicenter trials that can provide solid evidence across diverse populations. This evidence would facilitate translating research findings into practical applications, ultimately informing more effective diabetes management strategies.
- Fasting plasma glucose
  Fasting blood glucose (FBG) is a vital indicator in diagnosing and managing T2DM, with levels of 126 mg/dL or higher on two separate occasions serving as a diagnostic threshold. The mDiabetes system is a smartphone‐based platform that enhances diabetes management through integrated glucose monitoring, physical activity tracking, insulin management, and hypoglycemia prevention [13]. In an RCT with 191 participants, individuals were divided into four groups based on their antidiabetic treatment: Group A (lifestyle modification), Group B (oral medications with low hypoglycemia risk), Group C (oral medications with higher hypoglycemia risk), and Group D (insulin users). Blood glucose levels were measured using a Bluetooth glucometer, which provided real‐time feedback and tailored insulin dosing recommendations. Participants recorded their glucose levels daily during a 2‐week run‐in period before being randomized into either the mDiabetes or control groups using paper logbooks. After 24 weeks, those using the mDiabetes system achieved significantly lower FBG levels compared to the paper logbook group (*p* = 0.026), demonstrating how mobile health technologies can enhance glycemic control and aid daily self‐monitoring in managing diabetes. A novel strategy involved CGM paired with mobile technology to enhance diabetes management [14]. In this study, 68 participants aged 18–45 utilized an implantable glucose sensor connected to a mobile app, allowing both patients and healthcare providers to access retrospective health data. This integration facilitated real‐time data sharing, leading to a notable decrease in FBG levels compared to controls (*p* < 0.05).
  Exploring mHealth's versatility further, a three‐arm RCT evaluated different levels of intervention among 234 patients aged 19–74 [15]. Participants were divided into three groups: usual care (UC), mobile diabetes self‐care (MC), and MC enhanced by personalized physician feedback (MPC). All groups utilized glucometers connected to the iCareD mHealth system for self‐monitored blood glucose (SMBG) data transfer. While all groups showed significant reductions in FBG from baseline (*p* < 0.05), no meaningful differences were found between the intervention arms over the 26‐week study period. These results imply that while mHealth tools are effective in facilitating glycemic improvements, additional layers of personalization may not always yield further benefits in short‐term interventions.
- 2‐h postprandial glucose

Blood glucose is measured 2 h post‐meal in the 2‐h postprandial glucose test, which provides valuable insights into glycemic control and cardiovascular risk. Efforts to manage postprandial hyperglycemia have increasingly incorporated mHealth technologies. In Hangzhou, China, an RCT evaluated a mHealth management model that combined an implantable glucose sensor with a mobile application [16]. This system enabled real‐time glucose monitoring and personalized feedback from general practitioners (GPs). Over 4 weeks, the intervention group had notably lower 2hPP levels than those in the control group, with statistical significance (*p* < 0.05), demonstrating the potential of mHealth tools to enhance glycemic management through continuous monitoring and tailored interventions.

The 2hPP test is clinically important yet underutilized compared to FBG and HbA1c despite its role in assessing glycemic variability linked to complications. Integrating mHealth tools into diabetes care shows promise for managing postprandial glucose through continuous monitoring and real‐time feedback, empowering patients toward better control. However, challenges like scalability, long‐term adherence, and equitable access need addressing for widespread impact. Future research should expand multicenter trials to validate these findings and further support mHealth's role in diabetes care.

#### 3.1.2 Cardiovascular Risk Factors

- Blood pressure
  Diabetes and hypertension are strongly linked, with both conditions influenced by lifestyle factors and common risk factors. Elevated blood glucose levels can lead to vascular damage, increasing the risk of hypertension in diabetic patients. The Ondu VDC program was evaluated for managing blood pressure in 55 patients with T2DM [17]. This program combined a mobile app, remote coaching from Certified Diabetes Care and Education Specialists (CDCES), connected blood glucose meters, and real‐time continuous glucose monitoring (RT‐CGM) devices. Participants engaged with their care team at least once a week and received educational materials via the app. Participants used RT‐CGM devices (Dexcom G6) intermittently over 4 months, with an initial 20‐day deployment followed by a cycle of 10 days “on” and 11 days “off” for the remaining sensors. Participants used glucose information for guidance and tracking, helping them connect blood sugar levels to their diet and lifestyle to better manage diabetes. The study reported a significant reduction in systolic blood pressure (*p* = 0.04), while the reduction in diastolic blood pressure was not statistically significant (*p* = 0.48).
  In contrast, an RCT investigated the impact of a text messaging intervention on 126 low‐income Hispanic patients with uncontrolled T2DM [18]. Although participants received blood glucose meters, testing strips, and instructions, the intervention did not yield significant improvements in blood pressure or other secondary outcomes. This lack of significant results may stem from baseline blood pressure levels being close to target values and the study's primary focus on glycemic control rather than blood pressure management.
  The reviewed studies suggest that mHealth interventions, particularly those incorporating health coaching or user‐friendly mobile applications, may positively impact blood pressure management in diabetic patients. However, the variability in outcomes emphasizes the need for more comprehensive studies to explore these correlations further and validate the effectiveness of mHealth interventions on a larger scale.
- Lipid profile
  Lipid abnormalities, including elevated total cholesterol, LDL, and triglycerides, along with reduced HDL, are closely linked to glycemic control in T2DM. Since dyslipidemia significantly contributes to cardiovascular complications, effective lipid management becomes crucial for this population. The LIVA 2.0 eHealth lifestyle coaching program was evaluated for its effectiveness in managing T2DM in Denmark. The intervention was initiated by a 1‐h motivational interviewing session with a health coach, who provided continuous support throughout the program. Patients were given login credentials for the LIVA 2.0 app, allowing them to establish personalized goals for diet, exercise, and sleep. The health coach tailored initiatives based on participants' preferences and personal barriers [19].
  Patients recorded their daily progress and communicated directly with their health coach, who offered individualized online coaching weekly for the first 3 months and biweekly thereafter. This combination of digital tools and personalized coaching resulted in significant reductions in total cholesterol and LDL levels (*p* < 0.05), highlighting the effectiveness of lifestyle‐focused digital health solutions in improving lipid metabolism while allowing flexibility for patients to engage even when feeling unwell.
  The iCareD mobile app was assessed for enhancing lipid management among diabetic patients [15]. A total of 269 participants were allocated into three groups: usual care (UC) (*n* = 71), mobile self‐care (MC) (*n* = 79), and MC with healthcare provider feedback (MPC) (*n* = 84). After 26 weeks, the MPC group achieved a significant reduction in total cholesterol (12.4 vs. 3.2 mg/dL in UC; *p* < 0.01) and improvements in LDL cholesterol (*p* < 0.05).
  Collectively, these results illustrate the promise of digital health interventions in addressing dyslipidemia and improving cardiovascular outcomes for patients with T2DM. However, further large‐scale investigations are necessary to verify their durability and applicability across diverse healthcare settings and populations.
- BMI

The rising prevalence of T2DM is closely linked to increasing body mass index (BMI) and weight, making weight management a critical focus in diabetes care. Mobile health interventions have shown promise in addressing these issues, with some studies reporting significant improvements in BMI and weight loss.

In Denmark, the LIVA eHealth app provided an engaging platform for patients to manage their health [19]. Users set personalized diet, exercise, and sleep goals while receiving weekly coaching sessions through the app. The intervention group lost an average of 4.24 kg, whereas the control group lost only 1.52 kg, with BMI reductions of 1.40 vs. 0.51 kg/m². These results indicate the value of combining technology with customized coaching to achieve meaningful outcomes.

The Glycoleap program offered patients a mobile app, health coaching, educational materials, and practical tools like glucometers and wireless weighing scales [20]. Compared to the control group, participants lost an average of 2.3 kg, and 20% managed to reduce their weight by 5 kg or more from baseline. This underscores the potential of pairing practical resources with digital health tools to encourage sustainable lifestyle changes.

However, not all interventions were equally successful. The TIME program in Houston targeted low‐income Latino patients through community health worker support, telehealth consultations, and mHealth communication but failed to produce significant changes in BMI or weight compared to usual care [21]. This confirms that cultural and socioeconomic factors may influence the effectiveness of specific strategies.

These findings suggest that mHealth interventions, particularly those incorporating personalized coaching, goal‐setting features, and activity tracking, can significantly aid in managing BMI and weight among T2DM patients. However, variability in results across studies calls for further research to refine these approaches and address barriers, such as accessibility, usability challenges, and patient engagement. Modified solutions that consider diverse populations may enhance the impact of mHealth tools in diabetes care.

#### 3.1.3 Insulin Dosage

Three studies have investigated the My Dose Coach (MDC) application, a US Food and Drug Administration‐approved mobile application combined with a web portal to help users titrate basal insulin dosage [22, 23, 24]. The application recommends basal insulin injection doses based on personalized doctor prescriptions and patients' data, including SMBG and FBG recorded by a glucometer and incidence of hypoglycemic events. Based on the study, patients underwent observation for 3 and 6 months, 16 weeks, and 12 weeks. Results indicated a significant drop in HbA1c levels in two studies (*p* < 0.001 and *p* = 0.0388). All three studies reported achieving FBG targets in intervention groups compared with control groups, which was statistically significant. One study declared reaching the SMBG target of 90–130 mg/dL in 58.9% of the patients (*p* < 0.001). Two studies revealed that the intervention group had significantly higher basal insulin doses at study completion compared to the control group (*p* < 0.01 and *p* = 0.0011). In terms of frequency of MDC use, in the study organized by Unnikrishnan et al., participants were categorized into three subgroups: high‐usage group ( > 3 days per week), moderate‐( < 1– ≤ 3 days per week), and low‐( ≤ 1 day per week) usage groups. At the end of the study, the analysis showed a significantly higher increase in insulin dose for high usage compared to medium usage overall, and moderate and low usage when stratified by titration duration. None of the studies reported serious incidence of hypoglycemia among subgroups.

Due to the use of insulin in many T2DM patients, titration of basal insulin dosage has been arguable. The lack of information about other T2DM drug medications can be mentioned as a limitation in this field, which can be surveyed in future studies.

#### 3.1.4 Diabetes Complications

Elevated blood glucose levels are the hallmark of T2DM and are linked to various complications that can significantly impact health. These complications are categorized into macrovascular issues, such as ischemic heart disease and stroke, and microvascular problems, including neuropathy, nephropathy, and retinopathy. Additionally, acute complications like diabetic ketoacidosis and hypoglycemia can arise. The DialBetesPlus app, an upgraded version designed for diabetes self‐management, was evaluated in a study involving 126 patients with moderately increased albuminuria (UACR: 30–299 mg/g creatinine) [25]. Over 12 months, participants used the app to track daily metrics such as step counts, blood sugar, blood pressure, and body weight. The app provided individualized feedback and dietary advice based on meal photos submitted by users. Compared with the control group, the intervention group achieved a significant 28.8% drop in UACR (*p* = 0.029). Secondary outcomes included improvements in HbA1c (−0.32 points; *p* = 0.041) and HDL‐C levels (*p* = 0.041), although changes in estimated glomerular filtration rate (eGFR) were not statistically significant.

Interval walking was explored as a method to improve health outcomes in T2DM patients using smartphone technology [26]. Participants followed a 4‐month program with the InstaWalk app to complete interval training sessions thrice weekly, alternating between slow and fast walking while monitoring heart rates. Findings reflected significant decreases in albuminuria (*p* = 0.002) and leptin/adiponectin ratios (*p* = 0.01), with a near‐significant reduction in HbA1c levels (*p* = 0.09). This approach illustrates how combining physical activity with mHealth tools can yield measurable health benefits.

In addressing diabetic foot ulcers (DFU), researchers examined thermometry combined with mHealth reminders among 172 T2D patients at high risk for DFU [27]. Participants were divided into two groups: one using thermometry alone and another receiving weekly mHealth reminders alongside thermometry. While both groups received education on DFU prevention using TempStat, which detects temperature differences as early warning signs of ulceration, results showed a higher incidence of DFU in the intervention group (24.1%) compared to the control group (11.4%). This unexpected outcome suggests that mHealth reminders may not have been effectively implemented or sufficient to improve outcomes.

Despite the wide range of complications associated with T2DM, research on mHealth interventions targeting these issues remains limited. While some studies have begun addressing microvascular complications like nephropathy and neuropathy, their findings are still inconclusive. Additionally, although AI has been explored for managing T2DM retinopathy through smartphone applications, there is no direct evidence yet on how standalone mHealth tools impact retinopathy outcomes without AI involvement. Additional studies are required to fill these gaps and clarify how mHealth can effectively address both macrovascular and microvascular complications of T2DM.

### 3.2 Patient‐Reported Outcome (PRO)

Patient‐reported outcome (PRO) is defined as the patient's direct health status without a clinician's interpretation. PROs include a wide range of information, such as functional status, self‐care, self‐efficacy, self‐management, health‐related quality of life (HRQoL), and so forth. This information is usually collected through questionnaires or interviews based on the patient's perspective [28, 29]. Diabetes self‐care, encompassing dietary habits, physical exercise, medication adherence, and glucose monitoring, is critical for effective T2DM management. Mobile health interventions show promise in enhancing these practices, though outcomes remain heterogeneous. For instance, a mobile‐based educational program for elderly patients in Ahvaz significantly improved self‐care behaviors through structured online sessions and peer support via WhatsApp, with the intervention group reporting better self‐management than controls (*p* = 0.001) [30]. Utilizing the Information–Motivation–Behavioral Skills (IMB) model, a diabetes self‐management application effectively promoted improvements in patients' self‐care behaviors (*p* = 0.02) and social motivation (*p* = 0.05), though personal motivation and behavioral skills remained unchanged.

Beyond self‐care, HRQoL is a critical outcome in diabetes management. HRQoL assesses the impact of diabetes on patients' physical, emotional, and social well‐being. Studies have shown that patients with T2DM generally report reduced HRQoL compared to the general public, with factors such as diabetes‐related complications, age, gender, and socioeconomic status influencing these outcomes. Mental health is a significant component of HRQoL, as diabetes can impact psychological well‐being through chronic stress, anxiety, and depression. The Pack Health program combined one‐on‐one coaching with digital education, leading to a 5% improvement in mental health scores [31]. The P2E2T2 Program used nurse coaching and wearable technology to reduce depression severity significantly [32]. Smaller studies, such as one using Fitbit devices, also reported improvements in anxiety and depression scores, suggesting that self‐monitoring tools enhance psychological well‐being [33].

Besides the positive impact of digital health tools on the outcomes of diabetic patients, several significant barriers hinder the effective use of mobile health apps for diabetes self‐management, particularly among individuals with T2DM. Time and energy requirements are major concerns, as many patients perceive tracking health data and learning to use these apps as burdensome, which may deter initial engagement [34, 35]. Additionally, the lack of support from healthcare professionals plays a critical role; many patients feel that their providers do not actively encourage or recommend these technologies, leading to decreased motivation to adopt them [34, 35, 36]. Financial constraints also pose a significant barrier, as the absence of reimbursement from insurance companies for diabetes management apps makes patients hesitant to invest in these tools, especially if they do not see clear benefits [35, 36]. Furthermore, complexity and usability issues with some apps can frustrate users, particularly those with limited technical skills or older adults who may struggle with navigation and functionality [35]. Finally, limited awareness about the existence and benefits of these apps among potential users further limits their uptake [34].

Recent literature identifies several barriers to effectively utilizing mobile health applications for managing T2DM. These barriers encompass perceived time constraints, insufficient support from healthcare providers, financial limitations arising from a lack of reimbursement, app complexity, and a general lack of awareness among potential users. Addressing these challenges through comprehensive education initiatives, user‐centered app design improvements, and increased engagement from healthcare professionals is essential to enhance health outcomes.

## 4 Discussion

### 4.1 Clinical Outcomes vs. Patient‐Reported Outcomes

This review highlights the increasing importance of mHealth and digital health solutions in managing T2DM, reflecting a shift toward more patient‐centered, technology‐enabled care. The included studies can be grouped into two main categories: those focused on clinical outcomes and those addressing patient‐reported outcomes. Numerous studies demonstrated that digital interventions, such as smartphone apps, telemedicine platforms, and wearable devices can significantly improve glycemic control, as reflected by reductions in HbA1c and fasting blood glucose [13, 14, 15, 16, 17, 22, 28, 29, 30, 37, 38, 39, 40, 41, 42, 43, 44, 45]. Beyond glycemic indices, several studies also reported positive changes in cardiovascular risk factors, including blood pressure, lipid profiles, and BMI, indicating a broader cardiovascular benefit [14, 16, 22, 29, 30, 39, 40, 42, 43, 44, 45]. Importantly, some interventions addressed diabetes‐related complications, such as albuminuria and DFU, further supporting the clinical utility of these technologies [31, 32, 33]. The improvements observed are likely attributable to the real‐time feedback, personalized guidance, and continuous monitoring capabilities provided by digital platforms [14, 16, 29, 30, 39, 40]. A second group of studies focused on patient‐reported outcomes, including quality of life, self‐efficacy, treatment satisfaction, and diabetes‐related distress [21, 23, 24, 25, 26, 27, 46, 47, 48, 49]. Digital health interventions incorporating educational content, interactive features, and regular communication enhanced self‐care behaviors, improved adherence, and reduced psychological distress associated with diabetes [21, 23, 24, 47, 48, 49]. For example, mobile app‐based coaching and SMS reminders increased patient confidence in disease management and healthier lifestyle choices [23, 24, 25, 47]. Our study's findings agree with previous research, confirming that digital health technologies can effectively improve both metabolic parameters and patient‐centered outcomes. The magnitude of benefit often depended on the intervention's level of personalization, the intensity of support, and the duration of follow‐up [14, 16, 23, 29, 30, 47]. Interventions offering tailored feedback and ongoing professional or peer support generally yielded more sustained improvements, emphasizing the multifactorial nature of effective digital health strategies. These interventions include coaching applications which provide personalized behavioral support and motivation, CGM‐linked systems that enable continuous real‐time glucose monitoring, wearable devices tracking physical activity and biological indicators to encourage healthy habits, and educational platforms delivering structured diabetes self‐management content. Evidence suggests that combining coaching with CGM or wearable technologies enhances both clinical outcomes and patient‐reported measures more effectively than focusing on a single modality. This integrated approach addresses several aspects of diabetes management simultaneously, supporting better engagement and sustained benefits. Further comparative studies are warranted to evaluate the effectiveness and adaptability of these interventions across diverse patient populations [12, 15, 29, 30, 39, 40, 49]. The effectiveness of mHealth interventions in managing T2DM appears to vary across different patient populations and intervention modalities. Among older adults, mobile‐based educational programs delivered through common messaging platforms have demonstrated significant improvements in self‐care behaviors, glycemic control, and psychosocial factors [30, 33]. In contrast, individuals from rural or low‐income settings benefit from interventions that incorporate SMS messaging, community health worker involvement, and culturally adapted telehealth support, which effectively improve medication adherence, glycemic markers, and self‐management practices despite challenges related to technology access and literacy [18, 21, 34, 36]. For patients with obesity, interventions employing digital lifestyle coaching apps with goal‐setting features, remote monitoring using connected glucometers, and personalized feedback have resulted in substantial reductions in BMI, HbA1c, and body weight [19, 23, 33]. Moreover, interventions integrating CGM with mobile applications and personalized clinician feedback have shown greater efficacy in glycemic control compared to standalone self‐monitoring [10, 17]. These findings emphasize the importance of matching intervention types to patient characteristics and contexts, highlighting that multi‐component, personalized mHealth programs tend to achieve more sustained clinical and patient‐reported benefits across diverse populations. Several studies underscored the importance of sustained user engagement and seamless integration of digital tools into routine clinical practice [34, 35, 36, 46]. Barriers such as digital literacy, access to technology, and cultural adaptation were frequently cited, especially for older adults and underserved populations [34, 35, 36]. Addressing these challenges is crucial for maximizing the reach and effectiveness of mHealth interventions.

### 4.2 Short‐Term vs. Long‐Term Impacts

The length of which digital health interventions are used has a noticeable impact on their outcomes on T2DM. Many trials demonstrated that mHealth interventions can lead to significant improvements in clinical outcome, most notably glycemic control, self‐care behavior, and lifestyle modifications, within short‐term evaluation period, typically between 3 and 6 months [13, 14, 16, 17, 31, 42, 43, 45, 48, 50]. These early improvements are frequently attributed to increased user engagement, structured monitoring, and the novelty of incorporating mobile technologies into daily routines. Nevertheless, several studies indicated that this initial benefit can diminish over time as user motivation or adherence declines after the intensive intervention phase ends [23, 24, 31, 39]. Long‐term follow‐up studies, generally running for 12 months or more, have reported more durable effects, especially in key measures like HbA1c, medication adherence, and psychological well‐being [9, 10, 11, 14, 16, 18, 21, 23, 24, 25, 32, 39]. Persistent improvement in clinical and behavioral outcomes was often linked to continuous feedback, adaptive goals, and repeated educational reinforcement, rather than to the intensity or novelty of the digital platform alone. These findings underscore the importance of designing mHealth interventions with long‐term engagement strategies, frequent updates, and flexible goal‐setting to ensure enduring patient benefit. Overall, while most digital health tools deliver rapid and meaningful short‐term gains, durable success in diabetes management often requires ongoing effort and extended support throughout the course of the disease.

### 4.3 Standalone Digital Tools vs. Integrated Clinical Support

The framework through which digital health solutions are implemented plays a crucial role in T2DM management. Standalone technologies, such as mobile applications, SMS reminders, and web‐based platforms, have shown improvements in glycemic control, self‐care behaviors, and patient‐reported outcomes, especially when they incorporate user‐friendly interfaces, personalized notifications, and interactive educational features [13, 14, 15, 16, 17, 31, 37, 43, 45, 48]. These solutions promote patient independence and autonomy, making them highly effective for individuals with proper diabetes knowledge and strong motivation. However, the long‐term effectiveness of standalone tools may be limited by challenges, including reduced user engagement over time, technological difficulties, and the absence of ongoing external support [31, 39]. In contrast, digital interventions that are systematically integrated into standard clinical care, whether through collaboration among healthcare teams, integration with electronic health records, or continuous support from healthcare professionals, tend to produce more significant and sustained benefits [9, 10, 11, 14, 16, 18, 21, 23, 24, 25, 32]. Integrated programs facilitate consistent monitoring, timely treatment adjustments, and personalized feedback, as well as psychosocial support and peer interaction opportunities. This comprehensive approach supports accountability and provides tailored guidance, resulting in greater reductions in HbA1c and improved adherence to recommended lifestyle changes, particularly for patients who face challenges in self‐management or have lower motivation. The evidence strongly suggests that the most consistent and durable improvements in diabetes outcomes are achieved when digital health technologies are embedded within broader clinical management frameworks and involve active engagement from healthcare professionals [9, 10, 11, 14, 16, 18, 21, 23, 24, 25, 32, 45, 48].

### 4.4 Strengths and Limitations

A key limitation of this review is the exclusive use of the PubMed database, potentially causing selection bias and limiting the comprehensiveness of the evidence base. Google Scholar was also searched to supplement the PubMed results. Additionally, the review did not use a structured method to assess the risk of bias, which raises the likelihood of bias in study selection and interpretation. The included studies also exhibited heterogeneity in design, intervention strategies, and outcome measures, which may restrict the validity of the findings. Despite these limitations, this review has important strengths. Synthesizing evidence from a diverse range of studies and settings provides a comprehensive overview of both clinical and patient‐reported outcomes associated with digital health interventions in T2DM. This broad perspective enhances the relevance of the findings for future investigations, healthcare delivery, and the development of new digital health solutions. Future systematic reviews, including additional databases could further validate these findings.

## 5 Conclusion

Despite growing evidence supporting mHealth clinical utility among patients with T2DM, mobile health solutions still face implementation barriers, including data processing challenges and limited clinical integration. This review can serve as a valuable resource for guiding future studies and supporting the development of digital health applications tailored to the needs of individuals with T2DM. Future research should address current gaps by expanding data sources, performing rigorous risk of bias assessments, and evaluating digital interventions' prolonged efficiency and cost‐effectiveness across varied populations.

## Author Contributions

**Farnam Gohardehi:** data curation, formal analysis, investigation, validation, writing – original draft, supervision, writing – review and editing. **Behdad Ghanami:** conceptualization, investigation, validation, writing – original draft. **Melika Silakhor:** data curation, investigation, writing – original draft. **Pooria Sobhanian:** conceptualization, project administration, supervision, validation.

## Ethics Statement

All the researchers confirm that this study has been carried out per the Journal's Practice Guidelines on Publishing Ethics and has been performed ethically and responsibly, with no research misconduct. The article has not been published previously, and is not under consideration elsewhere.

## Conflicts of Interest

The authors declare no conflicts of interest.

## Transparency Statement

The lead author Pooria Sobhanian affirms that this manuscript is an honest, accurate, and transparent account of the study being reported; that no important aspects of the study have been omitted; and that any discrepancies from the study as planned (and, if relevant, registered) have been explained.

## Supporting information


**Table S1:** Search syntax to identify relevant documents.

## Data Availability

Data sharing is not applicable to this article, as no new data were created or analyzed in this study.
