# Supplementary material for: Digital Health Interventions for Type 2 Diabetes: A Narrative Review of Mobile Technologies and Their Impact on Patients' Outcomes
Source: Health Sci Rep. 2026 May 25;9(6):e72116. doi: 10.1002/hsr2.72116 (PMC13240062; doi:10.1002/hsr2.72116)
Supplement: Supplementary file 1 — Table S1: Search syntax to identify relevant documents. [file HSR2-9-e72116-s001.docx]

Supplementary:

Table S1. Search syntax to identify relevant documents

| Table S1. Search syntax to identify relevant documents | | |
| --- | --- | --- |
|  | PubMed | Google Scholar |
| Search strategy | ((Diabetes Mellitus, Type 2[tiab] OR NIDDM[tiab] OR Type 2 Diabetes Mellitus[tiab] OR Type 2 Diabetes[tiab] OR Diabetes, Type 2[tiab])) AND ((Telemedicine[tiab] OR Digital Health[tiab] OR Mobile Application[tiab] OR Mobile App[tiab] OR Mobile Phone[tiab] OR mHealth[tiab] OR Smartphone[tiab])) | ("Diabetes Mellitus, Type 2" OR NIDDM OR "Type 2 Diabetes Mellitus" OR "Type 2 Diabetes" OR "Diabetes, Type 2") AND ("Telemedicine" OR "Digital Health" OR "Mobile Application" OR "Mobile App" OR "Mobile Phone" OR mHealth OR Smartphone) |
| Publication Date | From 2015/01/01 to 2025/01/01 | From 2015/01/01 to 2025/01/01 |

PubMed Google Scholar

Search strategy ((Diabetes Mellitus, Type 2[tiab] OR NIDDM[tiab] OR Type 2 Diabetes Mellitus[tiab] OR Type 2 Diabetes[tiab] OR Diabetes, Type 2[tiab])) AND ((Telemedicine[tiab] OR Digital Health[tiab] OR Mobile Application[tiab] OR Mobile App[tiab] OR Mobile Phone[tiab] OR mHealth[tiab] OR Smartphone[tiab])) (“Diabetes Mellitus, Type 2” OR NIDDM OR “Type 2 Diabetes Mellitus” OR “Type 2 Diabetes” OR “Diabetes, Type 2”) AND (“Telemedicine” OR “Digital Health” OR “Mobile Application” OR “Mobile App” OR “Mobile Phone” OR mHealth OR Smartphone)

Publication Date From 2015/01/01 to 2025/01/01 From 2015/01/01 to 2025/01/01
